# Intraoperative In Vivo Imaging Modalities in Head and Neck Cancer Surgical Margin Delineation: A Systematic Review

**DOI:** 10.3390/cancers14143416

**Published:** 2022-07-14

**Authors:** Kurtis Young, Enze Ma, Sameer Kejriwal, Torbjoern Nielsen, Sukhkaran S. Aulakh, Andrew C. Birkeland

**Affiliations:** 1 John A. Burns School of Medicine, Honolulu, HI 96813, USA; kcyoung@hawaii.edu (K.Y.); enze@hawaii.edu (E.M.); sameerk@hawaii.edu (S.K.); nielsent@hawaii.edu (T.N.); 2 School of Medicine, University of California, Davis, CA 95817, USA; ssaulakh@ucdavis.edu; 3 Department of Otolaryngology—Head and Neck Surgery, University of California, Davis, CA 95817, USA

**Keywords:** fluorescence lifetime imaging, head and neck cancer, hyperspectral imaging, intraoperative imaging, narrow band imaging, near-infrared fluorescence, optical coherence tomography, otolaryngology, Storz Professional Image Enhancement System, surgical margin

## Abstract

**Simple Summary:**

The complete removal of cancerous tissue is an important predictor of patient outcomes after surgery, in particular for head and neck cancer surgery. Normally, surgical tissues are examined by a pathologist to determine whether a full removal of the cancer was performed. However, these analyses are often imperfect due to time constraints, limitations in frozen sections, and tissue orientation challenges. Newer intraoperative imaging techniques have shown great promise for increasing both the accuracy and efficiency of this process while in the operating room. This review summarizes and analyzes the current state of the literature on intraoperative imaging in determining cancer margins in head and neck cancers.

**Abstract:**

Surgical margin status is one of the strongest prognosticators in predicting patient outcomes in head and neck cancer, yet head and neck surgeons continue to face challenges in the accurate detection of these margins with the current standard of care. Novel intraoperative imaging modalities have demonstrated great promise for potentially increasing the accuracy and efficiency in surgical margin delineation. In this current study, we collated and analyzed various intraoperative imaging modalities utilized in head and neck cancer to evaluate their use in discriminating malignant from healthy tissues. The authors conducted a systematic database search through PubMed/Medline, Web of Science, and EBSCOhost (CINAHL). Study screening and data extraction were performed and verified by the authors, and more studies were added through handsearching. Here, intraoperative imaging modalities are described, including optical coherence tomography, narrow band imaging, autofluorescence, and fluorescent-tagged probe techniques. Available sensitivities and specificities in delineating cancerous from healthy tissues ranged from 83.0% to 100.0% and 79.2% to 100.0%, respectively, across the different imaging modalities. Many of these initial studies are in small sample sizes, with methodological differences that preclude more extensive quantitative comparison. Thus, there is impetus for future larger studies examining and comparing the efficacy of these intraoperative imaging technologies.

## 1. Introduction

A clear surgical margin is one of the strongest prognosticators in head and neck cancer (HNC) [1], and positive margins have been shown to drastically raise the rates of local recurrence and all-cause mortality in these cohorts [2,3]. Despite this understanding, surgical extirpation with clear margins can be particularly difficult to achieve in certain HNC subsites due to their close proximity to vital structures, irregular tumor invasion patterns, and occult or undetectable spread of disease. Indeed, the rate of positive margins in HNC is among the highest across cancers, ranging between 10% and 30% [4].

The current standard of care in HNC surgery often utilizes frozen section analysis for intraoperative margin assessment. However, histopathologic analysis of frozen sections for surgical margins is limited by time constraints, architecture distortion, and inability to assess certain tissues, with decreased accuracy pertaining to close and positive margins [5]. Furthermore, key histologic features, including lymphatic, vascular, and perineural invasion, and assessment of the true depth of invasion and invasive tumor fronts are limited with frozen sections, [6] and may not be known until permanent pathological sections after surgery. Additionally, in instances of positive frozen margins requiring intraoperative surgical re-excision, there exist significant challenges in tissue reorientation and identifying appropriate locations to re-excise, increasing the propensity for error and missed residual cancer [7]. Consequently, head and neck surgeons continue to be challenged in accurately identifying surgical margin features in the present day. This issue is of particular importance since the presence of positive margins can lead to substantial changes in treatment plans and survival outcomes [8,9].

Advances in intraoperative imaging techniques have been developed to address these limitations in determining cancer margins in HNC. These modalities include optical coherence tomography, narrow band imaging, autofluorescence, and fluorescent-tagged probe techniques. Initial studies have demonstrated encouraging sensitivity and specificity in detecting HNC margins and cancer from normal tissue. However, these studies remain preliminary and in smaller cohorts. Additionally, the different techniques have not been compared with one another. For these reasons, firm guidelines have yet to be established in the use of intraoperative imaging technologies, despite these initial promising results. To date, there have been no attempts to collate and compare these investigations pertaining to HNC in the literature. The current study aims to address these gaps in the literature by providing a comprehensive review of current intraoperative imaging modalities, with the goal of providing future directions and guidance on this topic to head and neck cancer surgeons.

## 2. Materials and Methods

**Figure 1:**
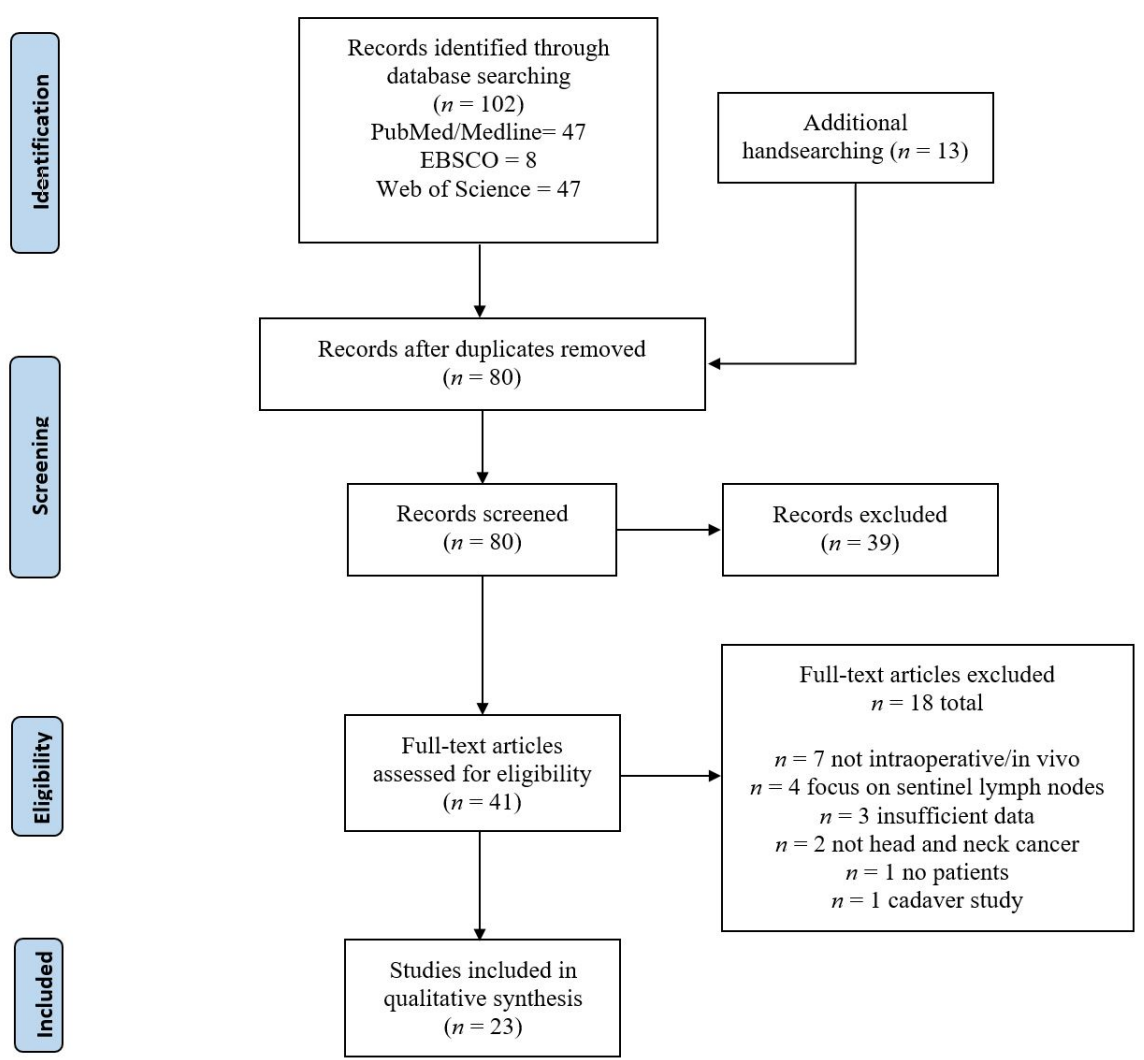
Flow diagram adapted from Preferred Reporting Items for Systematic Reviews and Meta-Analyses (PRISMA) 2020.

Author K.Y. conducted a comprehensive literature search through PubMed/Medline, CINAHL (EBSCOhost), and Web of Science, with no restrictions in publication date. Search terms including “head and neck”, “oral”, “pharyngeal”, “laryngeal”, “malignancy”, and “intraoperative imaging” were combined in differing permutations, as detailed in Appendix A. Duplicate studies were removed through the Rayyan QCRI reference management software, and the remaining titles and abstracts were independently screened by K.Y. and T.N. Author K.Y. subsequently conducted a full-text assessment of the screened studies. A number of studies were then identified through handsearching. The inclusion criteria were that the primary imaging modality was performed in an intraoperative, in vivo setting, with the primary outcome measure being tumor margin or malignant tissue differentiation. All non-English manuscripts were excluded from this review. Furthermore, animal and cadaver investigations were not included in this study. An abstraction form was created prior to data extraction. Authors E.M. and S.K. independently extracted data pertaining to study ID (publication dates, author names), study parameters (design, imaging modalities), and study findings (key outcomes, clinical significance and limitations). Author K.Y. independently verified all extracted data. This process is characterized in Figure 1.

## 3. Results

There were 102 titles initially identified through the systematic database search, with 67 remaining after duplicate deletion. There were 13 studies that were subsequently identified through handsearching. Seven studies were excluded for not featuring an intraoperative and in vivo procedure. Four studies were excluded for focusing on lymph node status instead of tumor or margin tissue differentiation. There were three studies excluded for having insufficient data or not involving an intraoperative or in vivo setting each. There were two studies that did not investigate head and neck cancers. The last study to be excluded was performed on cadavers. After study screening, 23 studies were included in this review. The technologies from the 23 studies included optical coherence tomography (2 studies), narrow band imaging (NBI) (3 studies), Storz Professional Image Enhancement System (SPIES) (3 studies), autofluorescence imaging (NBI) (4 studies), hyperspectral imaging (HSI) (1 studies), fluorescence-tagged imaging (5 studies), and tag-free fluorescence imaging (4 studies). One included study investigated both SPIES and NBI. Descriptive technical information on the included imaging modalities is shown in Table 1. All studies with sensitivities and specificities regarding malignant and benign tissue differentiation are characterized in Table 2.

### 3.1. Optical Coherence Tomography

Optical coherence tomography (OCT) measures the echo time delay and intensity of light, which is reflected and captured with low-coherence interferometry. OCT is a noninvasive and label-free diagnostic tool that can deliver high-fidelity imaging in real-time intraoperative settings, providing vital information on tumor margins. Since its inception in 1991, OCT was largely relegated to ophthalmological use [10]. However, technological advances over the past couple of decades have subsequently broadened the application of OCT across several disciplines, including in head and neck surgery [11]. This technology is comparable to that of ultrasound imaging, but OCT relies on captured data from reflected light instead of sound [12]. The first investigations utilizing OCT in head and neck tissues were conducted on the larynx, which was an optimal location due to its relatively thin epithelium [13,14,15]. In their prospective series with 33 subjects, Englhard et al. incorporated OCT technology together with surgical microscopy, allowing for a hands-free imaging process with improved surgical precision [16]. This investigation reported that microscope-integrated OCT imaging was able to correctly differentiate malignant (17/18) from benign laryngeal lesions (4/5), with poorer performance regarding premalignant processes (1/5). Nonetheless, this study also suffered from similar drawbacks including inferior image penetration and provided little insight regarding surgical margins. Indeed, the reported maximum penetration depth of 1.2 mm (average = 0.6 mm) poses potential limitations in the applicability of this imaging modality on bulkier, deeply invasive, or submucosal lesions and across other cancer subsites [15,16].

Nearly a decade later, this technique has been expanded to oral malignancies as well in intraoperative ex vivo settings [17,18]. Here, a malignant architecture detected by OCT was found to correspond to the histopathologic imaging counterparts with high sensitivity (81.5%) and specificity (87.0%) in detecting tumor margins [18]. In a recent investigation, Sunny et al. found an excellent concordance between OCT imaging and histopathologic analysis of oral cavity HNC, with a sensitivity and specificity both of 100% [19] (Table 2). This pilot study with 14 patients was the first to utilize in vivo OCT intraoperatively to delineate tumor margins in the oral cavity, suggesting the potential for equivalency of this methodology to permanent histopathological analysis. In summary, nevertheless, larger prospective studies are necessary to validate these findings.

### 3.2. Narrow Band Imaging

NBI utilizes an optical filter that only permits a narrow range of wavelengths to be emitted as light, with varying levels of tissue penetrance dependent on the selected spectra [20]. This technology highlights hemoglobin-carrying regions and relies on the presumption that cancers have higher density and aberrant vascularity. Although several prior investigations have confirmed the utility of NBI in screening for oropharyngeal and hypopharyngeal cancers [21,22], these data were acquired in an outpatient setting and were not used to guide intraoperative management. One of the first investigations utilizing NBI intraoperatively to determine surgical margins was conducted by Garofolo et al. in 2015 [23]. Here, 67 subjects with laryngeal lesions were intraoperatively assessed with NBI, and final pathological analysis yielded a 3.6% rate for positive margins compared with 23.7% for a historical control group. Furthermore, Klimza et al. reported that NBI had an accuracy and a sensitivity of 85.7% and 100%, respectively [24]. Although these findings were superior to that of white light alone, more information on specificity was unavailable due to the inclusion criteria of biopsy-proven cancer, precluding the identification of false-positive results. Piersiala et al.’s investigation mirrored these results in their sample of 98 patients with laryngeal lesions in 2018 [25]. Here, the authors performed cordectomies using a combination of white light and NBI endoscopy. One subset of 10 patients underwent NBI endoscopic imaging alone due to suspected peripheral margins, revealing several cases of moderate dysplasia (4), severe dysplasia (2), carcinoma in situ (3), and hyperkeratosis (1). The significance of these results was that these aberrant specimens were invisible on white light endoscopy, indicating a possible advantage of NBI in surgical margin detection. After final histopathological analysis, all tumor margins were determined to be clear. The investigators found an accuracy, sensitivity, and specificity of 99.0%, 100%, and 99.0%, respectively. Although these investigations were limited to neoplasms of the larynx, it is important to note that other limited studies across other HNC subsites have been performed [26]. However, extralaryngeal NBI imaging is often limited by the presence of lymphoid-tissue-dense regions, differing degrees of tumor thickness, and tissue keratinization [27]. While NBI shows promising results in surgical margin delineation, all studies were restricted by the need for an endoscopic camera for imaging. Larger controlled trials and prospective studies are required before formally incorporating NBI in the clinical setting.

### 3.3. Storz Professional Image Enhancement System

The Storz Professional Image Enhancement System (SPIES) utilizes a high-definition camera system with image-enhancing technology to improve the appearance of the mucosal surface and the vascular architecture across five spectral ranges [28,29]. This system also highlights contrast for vascular arrangements. Initial studies with SPIES found that it was comparable to NBI in the recognition and analysis of vascular patterns in typical benign and malignant lesions of laryngeal and hypolaryngeal pathologies [30]. Abdullah et al. investigated SPIES endoscopy with Ni et al.’s classification system for the detection of upper aerodigestive tract tumors and found a sensitivity and a specificity of 97.5% and 94.7%, respectively, regarding both benign and malignant lesions [31,32], enabling complete tumor resection by accurately delineating between healthy and tumorous tissue. This was found to be greater than the sensitivity and specificity of white light endoscopy, at 77.5% and 84.2%, respectively. 

Li et al. conducted a pilot study involving SPIES technology in the assessment of sinonasal inverted papilloma (SIP) [33]. The study involved a total of 115 patients, including 80 patients with SIPs or nasal polyps, and 35 healthy controls. Of the 80 patients, 44 patients were found to have nasal polyps, and 36 were found to have SIPs on histopathologic examination. White light endoscopy successfully detected 41 of the 44 cases with nasal polyps and 24 of the 36 cases with SIPs. Using SPIES endoscopy, 43 of the 44 cases with nasal polyps and 33 of the 36 cases with SIPs were successfully identified. The authors reported a sensitivity and a specificity of 91.7% and 95.5%, respectively. The results of this study further demonstrate SPIES as a rapid and noninvasive, accurate, real-time modality that can be used to detect SIPs. Englhard et al. conducted another study using SPIES to detect nasal and paranasal sinus diseases intraoperatively with the objective of evaluating its feasibility in clinical practice [34]. Twenty-seven patients with varied pathology and 10 healthy individuals were examined with both SPIES and white light endoscopy. Two questionnaires were provided: the first evaluated the surgeon’s subjective experience with SPIES technology; the second evaluated whether specific advantages exist between SPIES and white light endoscopy. Results of the study show that SPIES subjectively facilitated the assessment of tumor extension, particularly in vascularized tumors, and it proved to be superior to white light endoscopy via the results of the questionnaires in the study. However, due to the limited nature of these investigations, further studies with randomization will be needed to determine the potential of integrating SPIES into clinical practice. The aforementioned studies thus far are better characterized in Table 3.

### 3.4. Autofluorescence Imaging

Fluorescence lifetime imaging (FLIM) has been a burgeoning area of research since it was first discovered in decades prior [35]. Here, endogenous fluorophores are excited by a pulsed laser, and subsequent fluorescent lifetimes are measured and quantified. One of the first intraoperative applications of this technique in HNC was performed by Sun et al., where an endoscopic apparatus was utilized to measure in vivo autofluorescence [36]. Here, FLIM was able to identify different patterns of intensity and lifetime between cancerous tissue, margin, and normal tissue, demonstrating the potential of this modality to be utilized in the intraoperative setting for oral cancers. However, this preliminary study was limited by a small sample of 10 patients and slow image capture rates. FLIM was further investigated in 10 patients undergoing transoral robotic surgery (TORS) at this same institution, although information regarding margins was not available since all tumor beds were clear of residual disease [37]. More recently, the same researchers conducted a larger prospective study using either TORS or an endoscope FLIM scanning method with 53 subjects diagnosed with either oral or oropharyngeal HNC [38]. Similar to the aforementioned studies, cancer tissues were found to have significantly weaker spectral intensities and shorter lifetimes in comparison with their healthy counterparts. The sensitivity (86%) and specificity (87%) of free-handed FLIM in differentiating malignant from healthy tissues were excellent, and remained high when challenged with point-measurement classifier outputs. While all of these findings are founded on studies with limited sample sizes, the potential for FLIM in the intraoperative identification of tumor margins is encouraging.

Following a similar fluorophore-dependent mechanism as FLIM, dynamic optical contrast imaging (DOCI) was developed to bypass the complex mathematical models required in FLIM [39]. This method offers shorter imaging time frames while still producing scalable, proportionally accurate fluorescence lifetimes. In a preliminary ex vivo study with 81 patients with primary hyperparathyroidism undergoing parathyroidectomy, DOCI was able to clearly differentiate the parathyroid glands from surrounding tissues [40]. When DOCI was applied in a smaller in vivo intraoperative setting for HNC, researchers were able to clearly differentiate malignant from healthy tissues through measuring fluorescent lifetimes [41]. However, this study did not quantify fluorescent intensity as was done in the aforementioned FLIM studies. The literature regarding this technique is still limited, and additional studies on the applicability of DOCI in HNC must be performed to accurately gauge its potential role in defining surgical margins.

### 3.5. Hyperspectral Imaging

Although first developed in the context of improving space exploration [42], HSI has been readily adapted to head and neck surgery. This methodology collects reflected light across a continuum of spectral bands, generating objective surface analysis data in a noninvasive, tracer-free process [43]. Halicek et al. were among the first to utilize this imaging modality to determine cancer margins in ex vivo HNC specimens [5,44,45]. In an ex vivo study of 102 patients with oral cavity HNC, these investigators compared the accuracy between HSI, autofluorescence, 2-deoxy-2-[(7-nitro-2,1,3-benzoxadiazol-4-yl) amino]-D-glucose (2-NBDG), and proflavin dye [5]. Here, HSI and autofluorescence demonstrated greater accuracy than when compared with their dye-based counterparts. However, while HSI was found to be more accurate in detecting cancer margins in conventional squamous cell HNC across nearly all contexts, autofluorescence proved to be superior in most aspects of HPV-positive HNC margin detection. In a smaller in vivo experiment of 24 subjects utilizing HSI imaging coupled with a 3D reconstructive algorithm, the accuracy, sensitivity, and specificity in determining tumorous tissues against healthy samples were found to be 81.3%, 83.3%, and 79.2%, respectively [46]. Similarly with other in vivo studies, limitations of this investigation included motion artifact and image noise from unamenable causes, such as patient pulse. Despite the clear promise of hyperspectral imaging in the intraoperative setting, more extensive prospective trials are needed to confirm these initial findings.

### 3.6. Near-Infrared Fluorescence

Near-infrared (NIR) fluorescent probes have been developed with the goals of improving cancer detection and characterization, lymphatic imaging, and intraoperative surgical guidance [58]. Near-infrared light (650–900 nm) has a deeper tissue penetration than visible-range light, making it a favorable imaging agent to guide tumor resection [58]. Furthermore, there are available surgical instruments allowing for the intraoperative detection of NIR fluorescence. Since tissues absorb and emit light at different wavelengths, surgeons can use this imaging modality to help differentiate between normal and cancerous mucosa based on fluorescence patterns [51]. The two major forms of NIR fluorescence, fluorescence reflectance imaging (FRI) and tomographic fluorescence imaging, each have unique properties [58]. While FRI displays high spatial resolution, cost and time efficiency, portability, and color flexibility, the tomographic fluorescence imaging can create three-dimensional images that have greater depth sensitivity but lower spatial resolution [58]. 

Several studies have investigated the use of label-free NIR fluorescent dyes in the delineation between cancerous and healthy tissues. Stubbs et al. explored how free indocyanine green (ICG) dye could be infused 24 h prior to surgery and still provide benefit to surgeons in 3/14 surgeries for squamous cell carcinoma and adenoid cystic carcinoma [47]. However, Scott-Wittenborn et al. noted that untagged ICG NIR fluorescence failed to help surgeons differentiate between cancerous and normal mucosa for 6 patients with oropharyngeal squamous cell carcinoma [48]. These findings could be attributed to poor fluorescence tumor target and greater vasculature for the oropharyngeal region. Despite this, in a separate investigation comparing the off-line analysis of ICG NIR imaging with histopathologic results in various mucosal HNCs, the sensitivity and specificity for malignant tissue were found to be 90.5% and 90.9%, respectively [49]. In regard to oral cavity HNC, another clinical trial of 20 patients discovered 4 tumor beds with abnormal fluorescence, leading to the excision of 2 additional pathology-confirmed residual malignant specimens [50]. However, the authors draw attention to an important caveat: inflammatory processes, regardless of etiology, may influence dye uptake, leading to increased chances of false-positive results.

### 3.7. Near-Infrared Fluorescence (Tagged Probes)

NIR fluorescently labeled probes targeting EGFR, which is overexpressed in the majority of HNC, have been investigated as an intraoperative imaging technique for HNC [51]. Van Keulen et al.’s study found that NIR fluorescently guided surgery using anti-EGFR antibodies conjugated to a NIR probe (panitumumab-IRDye800CW) helped improve surgeon decision making for 3/14 head and neck squamous cell carcinoma resections [51]. Additionally, 10/10 patients had deep margins that were negative for fluorescence, with all final pathologic specimens demonstrating clear tumor margins >3 mm. The same researchers conducted another study with the same tagged anti-EGFR antibodies with 20 patients with HNC and demonstrated that in situ tumors were associated with higher fluorescent intensities than healthy tissue, regardless of age, sex, tumor site, or size [52]. Furthermore, fluorescent intensities were not significantly altered by ambient lighting or variations in EGFR expression levels, giving encouragement to the further investigation of fluorescently labeled probes targeting EGFR as a valuable surgical imaging modality in HNC. However, it should be noted that Zhou et al. reported that cellular EGFR expression, tumor cell density, and plasma antibody concentrations can affect the distribution of tissue fluorescence [53]. Additionally, the authors reported a sensitivity and a specificity of panitumumab-IRDye800 NIR imaging in detecting malignant tissues in the head and neck at 97% and 86% (Table 2), respectively. Several fluorescent nanoprobes have also been developed to rapidly react to acidic environments encountered in malignancy-associated metabolic acidotic states, releasing conjugated ICG dye [54]. An investigation by Voskuil et al. identified tumor-positive margins with 100% sensitivity [55]. However, the specificity for HNC was unclear due to the pooling of findings across different cancer types. However, similarly to their tag-free counterparts, the use of these specific acid-activated probes may be complicated by inflammatory processes.

An important drawback of both free and tagged NIR fluorescence imaging is the requirement of fluorescent tracers and associated probes, which are associated with their own risks [56,57]. Furthermore, the use of these fluorescent dyes requires a separate preoperative injection, which may need to be completed several days in advance. Although many advances with NIR have been made for head and neck cancers, further research with larger sample sizes looking at the benefits of this imaging for patient survival must be conducted. The modalities from Section 3.4, Section 3.5, Section 3.6, Section 3.7 are better characterized in Table 4.

## 4. Discussion

This current investigation has identified and collated the available literature on the use of intraoperative in vivo imaging modalities in head and neck surgery. While the reported sensitivities (83.0–100%) and specificities (79.2.0–100%) were promising across the included studies, these data were derived from preliminary investigations with small cohorts examining specific anatomical subsites. Subsequently, additional prospective trials with greater sample sizes must be performed to acquire an accurate assessment of sensitivities and specificities of the aforementioned modalities. These investigations should also attempt to compare and contrast cohorts with different HNC sites to determine the generalizability of these modalities across different subsites. 

Notably, the integration of such technologies may not be feasible outside of larger academic institutions. Several fluorescent materials may be more accessible than others, including more commonly used nontagged fluorescent molecules (ICG) when compared with antibody-tagged fluorescent probes (panitumumab-IRDye800). As these agents are investigational, they currently require clinical trial designs and appropriate personnel to run such trials, which carry significant cost and effort. Furthermore, tools with high overhead costs, including the pulsed diode lasers used in FLIM/DOCI or hyperspectral cameras required in HSI, currently are not available in rural areas or developing countries. Therefore, these imaging modalities may not be of immediate benefit to those with limited access to high-quality healthcare, regardless of any potential benefits that they may confer. Further considerations to expand access to potential care-altering technologies should be made in the additional expansion of intraoperative imaging devices. Furthermore, many of these technologies have been seamlessly integrated into existing clinically approved devices, including TORS, endoscopes, and various cameras. In these cases, added time to surgery is often minimal (a few minutes at most) in comparison with the overall surgical length, which can last many hours. However, the added costs of these technologies, at least in the early investigational stage, may be high and may not be amenable to those in developing or under-resourced regions. 

While several of the aforementioned modalities provide high-resolution imaging of tissue architecture, those relying on light penetrance for image acuity suffer from various drawbacks. In the case of OCT, the quality of the image is dependent on the degree of multiple scattering in a sample, preventing meaningful imaging at deeper tissue depths. While the conventional wavelength of light used with this modality is around 1.3 µm, there have been several studies that have utilized longer wavelengths (1.7 µm) of light with improved imaging acuity at deeper depths [59,60]. Additionally, newer light sources utilizing vertical-cavity surface-emitting lasers (VCSELs) have been shown to provide an imaging depth of up to 38 mm, but the clinical utility of this option has yet to be confirmed [61]. NBI, SPIES, and hyperspectral imaging are similarly limited by the penetrance of emitted light, which may be distorted by blood, mucus, or inflammation. However, these problems may be avoided by maintaining good technique, with the prompt evacuation of mucus and prevention of bleeding. Additionally, good visualization of the tumor margins is often performed prior to the initiation of surgical resection and alteration of the mucosal vascular network; further challenges and assessment of using these technologies to assess deep and postresection margins will be critical to adding utility for these devices. 

Despite showing great promise, FLIM autofluorescence imaging has been limited by time-consuming, computationally intensive models, and this is particularly true with lower photon/pixel ratios [62]. However, the field of autofluorescence imaging is rapidly evolving, with newer techniques including DOCI being developed to overcome these challenges with prompt image acquisition. Several frameworks making use of deep learning methods have been developed to achieve rapid fluorescence lifetime computation. For instance, artificial neural networks (ANN) have been used in conjunction with FLIM to enhance computational times 180-fold without decreases in performance when compared with conventional least squares estimation (LSE) [63]. Similarly, convolutional neural network (CNN) FLIM models have been demonstrated to be superior to LSE in terms of both accuracy and speed [64]. However, these methods are similarly ineffective in the low-photon-count settings. Most recently, a new deep-learning-based model, flimGANE, has been designed to overcome this obstacle. This new modality functions at 258-fold the speed of LSE and impressively 2800-fold faster than the gold standard maximum likelihood estimation (MLE) model. Most importantly, flimGANE has been shown to perform consistently with a photon-count-to-pixel ratio of 50, which is half of what is typically required in MLE [62]. Furthermore, the incorporation of augmented reality with FLIM represents another exciting area in which this field is moving towards, showing promising results in head and neck surgery [65]. In augmented or mixed-reality paradigms, overlaid tumor information from FLIM or other similar technologies could in theory enhance surgeon decision-making real time while not needing to look away from the operative field. The rapid progression observed in FLIM, and other autofluorescence-derived modalities is encouraging, yet these newest developments have yet to be rigorously trialed in the operating room. 

Tag-free fluorescent dyes have shown some initial findings in surgical margin demarcation due to the increased vascular supply observed in malignant tissues. Unfortunately, due to the lack of specificity of these dyes, other sources of inflammation or tissue damage may limit their efficacy and accuracy as diagnostic agents. Indeed, nanoparticle distribution in the context of systemic shock was found to be heavily retained in various organs, including the spleen [66]. Increased vascular permeability and subsequent dye uptake in nontarget tissues or organs in such states may not be uncommon in patients with chronic, advanced malignant processes [67]. Fluorescent-tagged targeted probes may provide an additional benefit in terms of localization to cancerous tissues in comparison. For instance, fluorescently labeled EGFR antibodies have been found to be highly selective and specific in surgical margin delineation for oral cancers [53]. However, targeted ligand expression can be variable across HNC, and there is currently no ubiquitous ligand across all HNCs. Further research on other probes and antigens should be conducted for additional HNC subtypes and HNC with different molecular expression patterns. For instance, aberrancies in HPV-associated or EBV-associated malignancies are dramatically different from nonviral HNCs [68,69], where EGFR expression is not normally elevated, but viral and other molecular aberrancies are present. 

Further technological iterations of imaging modalities will be critical to improve the sensitivity and specificity of cancer imaging agents. For instance, recent advances in near infrared imaging have been made in NIR-II models (with wavelengths ranging from 1000 to 1700 nm), which have advantages over NIR-I, including deeper tissue penetration and an improved signal-to-noise ratio [70]. Therefore, there have been considerable recent efforts to develop new probes that emit in the NIR-II window, with implications as a highly sensitive and specific surgical imaging agent. However, these newer probes have yet to be registered under any clinical trials, and current research continues to be preliminary.

Additionally, several of the aforementioned imaging modalities have already been combined as multimodal options. For instance, Vasquez et al. combined FLIM, OCT, and Raman spectroscopy in a clinically applicable and compact setup [71]. Here, the authors recommended utilizing the faster imaging modalities (FLIM and OCT) first, followed by Raman spectroscopy to examine areas of interest more closely. It is important to note that while Raman spectroscopy and Fourier-transform infrared spectroscopy have been trialed in the early diagnosis of HNC, there have been no corresponding intraoperative in vivo studies measuring surgical margin delineation [72]. However, these technologies would be of high interest in future investigations focused on assessing surgical margins. In another investigation by Nothdurft et al., FLIM was combined with the NIR fluorescent dyes, cypate and 3,3-diethylthiatricarbocyanine iodine [26]. However, this methodology was limited by the photostability of organic NIR dyes, which often caused the photobleaching images at higher resolutions. While several other modalities have been combined in various permutations, the vast majority of these trials were not performed in an in vivo setting. Regardless, it is possible that novel combinations of imaging modalities will supersede current single-modality imaging techniques in the future. There may be opportunities in re-evaluating previously tested imaging agents in such combinations, such as 5-aminolevulinic acid (5-ALA), which has been investigated as a topical visualization agent [73] with some initial encouraging results, but with significant limitations, including phototoxicity, bronchospasm, and poor specificity under specific circumstances [74]. In such combinations, limitations of one agent may be overcome (e.g., lower dose for 5-ALA to limit toxicity, or combining FLIM with NIR-tagged antibodies to improve sensitivity and specificity). Further research will need to be conducted on the safety and efficacy of such multimodal methods before their routine intraoperative implementation.

Although the featured imaging modalities have varying benefits and drawbacks, the overarching important points are outlined as follows. The major strength of intraoperative in vivo imaging is providing real-time, high-fidelity delineation of the surgical margin, which is integral in improving patient outcomes. However, the major weaknesses are that many of these studies are still in their preliminary stages, and the required technologies may not be available in rural or developing areas. Further research will create opportunities to determine the optimal imaging modality for surgical margin detection, with improvements in efficiency and accuracy. However, major threats on the implementation of these technologies include the inherent difficulties of running surgical clinical trials, precluding meaningful comparisons between imaging modalities from being made. Encouragingly, the increasing number recently completed, active, and pending clinical trials that are using these technologies (e.g., EGFR antibodies tagged with fluorophores: NCT02415881, free ICG: NCT03745690) highlights the burgeoning interest in this field. Nevertheless, larger multi-institutional studies with standardized data acquisition and analysis should be conducted for future investigation and meta-analysis.

## 5. Conclusions

This study is the first attempt to gather and synthesize the current literature on using intraoperative imaging to define surgical margins in the context of HNC. Head and neck surgeons will likely have access to a broader array of imaging options as these techniques continue to develop and improve throughout the years to come. Although many of these imaging modalities are promising, all of the included investigations have been limited by smaller cohorts and differences in measures, control groups, and institutional heterogeneity. Additional iterative studies with improved sensitivities/specificities are necessary to build on the existing data and should explore the potential of multimodal processes. Future research should feature larger controlled trials and strive to systematically report findings for comparison in this burgeoning, critical, and exciting field.

## Figures and Tables

**Table 1:** Imaging modality properties.

Imaging Modality	Mechanism	Strengths	Weaknesses
Optical coherence tomography [10,11,12,13,14,15,16,17,18,19]	Measures the echo time delay and intensity of light, which is reflected and captured with low-coherence interferometry. This measurement is compared with that of a predetermined reference path length to generate the image.	Radiotracer-free, high-resolution, rapid image acquisition has been integrated on microscopes.	Limited depth by light penetrance, imaging quality may be often limited by optical scattering from blood vessels.
Narrow band imaging [20,21,22,23,24,25,26,27]	Uses special filters that force the emission of narrow wavelengths of light. These wavelengths (usually between 440 and 560 nm) are more readily absorbed by hemoglobin, leading to a higher contrast of blood vessels along the surface mucosa.	Radiotracer-free, rapid switch between white light and NBI, readily applied to endoscopes/cameras.	Presence of blood and mucus may interfere with imaging, inflammatory changes may be misinterpreted as dysplasia.
Storz Professional Image Enhancement System [28,29,30,31,32,33,34]	Utilizes several modes, including spectra A and spectra B, which are differentiated by separate color filters for detecting vascular arrangements. Additionally, the Clara and Chroma modalities alter the brightness of an image, leading to improved anatomical contrast, particularly regarding darker spots.	Radiotracer-free, several different filters/modes to select from, readily applied to endoscopes/cameras.	Similar weaknesses as NBI (mucus, inflammation, bleeding), with comparable results and costs despite increased complexity.
Fluorescence lifetime imaging [35,36,37,38]	Excites endogenous fluorophores with pulsed laser; subsequent fluorescent lifetimes from photon emissions are measured and quantified. The half-life and intensity of the resulting emission can be compared between tissues of different types.	Radiotracer-free autofluorescence-guided, readily applicable to endoscope/camera, minimally affected by nonuniform illumination or absorptive mediums (blood).	Prolonged scan time due to laser technology, point-scanning, off-line image data processing, and complex mathematical processes, need for validated database of FLIM features confirmed through histopathology.
Dynamic optical contrast imaging [39,40,41]	Utilizes similar fluorophore-dependent mechanism as FLIM but utilizes a unique methodology in data processing that allows for the summation of pixel distributions that are proportional to the actual measured fluorophore activity.	Similar benefits as FLIM but with shorter imaging times.	Very limited testing completed in head and neck cancers, no side-by-side comparison with other modalities.
Hyperspectral imaging [5,42,43,44,45,46]	Makes use of extended spectral information from tissues, outside the limited range of RGB wavelengths. This allows for the generation of a 2D image with a corresponding 3D dataset on wavelengths (hyperspectral cube).	Radiotracer-free, provides valuable data to the granularity of cell nuclei, rapid image acquisition (seconds).	Limited by motion artifact, blood flow/oxygenation, saliva/mucous, complex analysis that cannot be performed normally by physicians.
Near-infrared fluorescence (tag-free) [47,48,49,50]	Unbound fluorescent dyes. This method depends on the increased vascularity of malignant tissues, leading to increased fluorescence of the targeted region of interest. Near-infrared light is used due to its greater tissue penetration.	Modern radiotracers rarely result in serious adverse effects.	Dye dependent, must preinject tracer and wait for distribution in targeted tissue, nonspecific dye distribution potential.
Near-infrared fluorescence (tagged probe) [51,52,53,54,55,56,57]	Fluorescent dyes are conjugated with probes (oftentimes antibodies). These probes either target specific antigens (e.g., EGFR) or are activated under specific environments (metabolic acidosis), allowing for more specific identifications of target tissues.	Targets tissue of interest with specific ligands, tagged fluorescence tumor-to-background ratio was consistent regardless of receptor (EGFR) density.	Certain probes may cause adverse effects not typically encountered with untagged fluorescent dyes, variability in ligand expression may limit probes.

**Table 2:** Studies reporting sensitivity and specificity in delineating cancerous from healthy tissues.

Ref.	Year	Author	Imaging Modality (In Vivo, Intraoperative)	n	Neoplasm Site	Sensitivity	Specificity
[19]	2019	Sunny et al.	Optical coherence tomography	14	Oral cavity	100.0%	100.0%
[25]	2018	Piersiala et al.	Narrow band imaging (NBI)	98	Larynx	100.0%	99.0%
[24]	2019	Klimza et al.	Narrow band imaging (NBI)	44	Larynx	100.0%	-
[30]	2018	Staníková et al.	Narrow band imaging (NBI)	73	Larynx, hypopharynx	83.0%	98.0%
			Storz Professional Image Enhancement System (SPIES)			86.0%	96.0%
[31]	2020	Abdullah et al.	Storz Professional Image Enhancement System (SPIES)	59	Larynx, nasal cavity, nasopharynx, oral cavity, oropharynx	97.5%	94.7%
[33]	2021	Li et al.	Storz Professional Image Enhancement System (SPIES)	115	Sinonasal	91.7%	95.5%
[38]	2020	Marsden et al.	Fluorescence lifetime imaging	53	Oral, oropharynx	86.0%	87.0%
[46]	2022	Eggert et al.	Hyperspectral imaging	98	Oropharynx, larynx, hypopharynx	83.3%	79.2%
[49]	2016	Schmidt et al.	Near-infrared fluorescence (tag-free)	55	Oral cavity, larynx, oropharynx, hypopharynx	90.5%,	90.9%
[53]	2022	Zhou et al.	Near-infrared fluorescence (tagged probe)	31	Head and neck, high-grade glioma, lung adenocarcinoma	97.0%	86.0%

**Table 3:** Studies from optical coherence tomography to the Storz Professional Image Enhancement System.

Ref.	Authors (Year)	Study Design (n)	Site	Imaging Modality of Interest	Key Findings/Outcome Measures	Clinical Significance
[16]	Englhard et al. (2017)	Prospective (28)	Larynx	Optical coherence tomography (OCT)	- Benign lesions: 17/18, premalignant lesions: 1/5, malignant lesions: 4/5 - 76% of laryngeal lesions were correctly identified	OCT was able to differentiate malignant from benign lesions
[19]	Sunny et al. (2019)	Prospective (14)	Oral cavity	Optical coherence tomography (OCT)	- 100% sensitivity and specificity in determining malignancy fields/margins - Excellent concordance between OCT and corresponding histopathologic analysis	Landmark study demonstrating the potential of OCT in in vivo imaging
[24]	Klimza et al. (2019)	Prospective (44)	Larynx	Narrow band imaging (NBI)	- White light sensitivity; specificity and accuracy were 79.5%, 20%, and 71.1% compared with that of NBI at 100%, NA, and 85.7%, respectively	NBI was superior to white light alone in detecting glottic cancers
[25]	Piersiala et al. (2018)	Prospective (98)	Larynx	Narrow band imaging (NBI)	- Intraoperative use of NBI improved % of negative margins in moderate advanced laryngeal cancer and supported the decision-making process during surgery in 9 of 10 cases	NBI can reduce the chance of positive margins for laryngeal cancers
[23]	Garofolo et al. (2015)	Prospective (82)	Larynx	Narrow band imaging (NBI)	- The rate of positive superior margins (23.7%) in historical control groups was higher than in the NBI group (3.6%) - There were 70 patients with negative margins, but 7, 2, and 3 positive deep, close, and positive superficial margins, respectively	NBI may increase the accuracy of detecting glottic cancers during early stages
[30]	Staníková et al. (2018)	Prospective (73)	Larynx and hypopharynx	Storz Professional Image Enhancement System (SPIES) and narrow band imaging (NBI)	- Benign lesions were histologically confirmed in 26 cases, and identified by both NBI and SPIES in 20/26 cases - The sensitivity and specificity of SPIES in the correct identification were 86% and 96.0%, respectively. The sensitivity and specificity of NBI was 83.0% and 98%, respectively.	NBI and SPIES are comparable in the detection of pathology in larynx and hypopharynx
[34]	Englhard et al. (2022)	Prospective (27)	Sinonasal	Storz Professional Image Enhancement System (SPIES)	- SPIES improved visualization, differentiation, and evaluation of the vascularization of paranasal pathologies, allowing for precise and accurate procedures	SPIES is a promising adjunct tool to evaluate nasal pathologies intraoperatively, especially in vascularized tumors
[33]	Li et al. (2021)	Prospective (115)	Sinonasal	Storz Professional Image Enhancement System (SPIES)	- Of the 80 patients, 44 patients were found to have nasal polyps, and 36 were found to have SIPs on histopathologic examination - SPIES detected 43/44 cases of nasal polyps and 33/36 cases with SIPs. Sensitivity was 91.7%; specificity was 95.5%	SPIES is a rapid and noninvasive, accurate, real-time modality that can be used to detect SIP against normal tissues
[31]	Abdullah et al. (2020)	Prospective (59)	Larynx, nasal cavity, nasopharynx, oral cavity, oropharynx	Storz Professional Image Enhancement System (SPIES)	- SPIES has a sensitivity and a specificity of 97.5% and 94.7%, respectively, which were greater than the sensitivity and specificity of white light endoscopy, found to be 77.5% and 84.2%, respectively	SPIES can be used in the detection of upper aerodigestive tract tumors, promoting early diagnosis and accurate margin delineation

**Table 4:** Studies from fluorescence lifetime imaging to near-infrared fluorescence imaging.

Ref.	Authors (Year)	Study Design (n)	Site	Imaging Modality of Interest	Key Findings/Outcome Measures	Clinical Significance
[38]	Marsden et al. (2020)	Prospective (53)	Oral cavity and oropharynx	Fluorescence lifetime imaging (endoscopic and TORS)	- Sensitivity and specificity for differentiating healthy from cancerous tissues were 86% and 87%, respectively, dropping to 72% and 69%, respectively, for point measurements - Cancer tissues were found to have significantly shorter lifetimes and weaker intensities	Cellular dysplasia was found at tumor margins, signifying that FLIM can detect the gradient between healthy and cancerous tissues
[36]	Sun et al. (2013)	Prospective (10)	Oral cavity	Fluorescence lifetime imaging (endoscopic)	- FLIM was able to identify different patterns in tumor, margin, and normal tissue regarding signal intensity and lifetime	Findings suggest possible use in determining surgical margins intraoperatively
[37]	Weyers et al. (2019)	Prospective (10)	Oropharynx	Fluorescence lifetime imaging (TORS)	- 9/9 preresection scans were able to show difference in at least one FLIM parameter between healthy and cancer cells	FLIM delineated cancerous tissues in the oropharynx and was more effective in vivo
[41]	Tajudeen et al. (2017)	Prospective (15)	Head and neck (cutaneous and mucosal)	Optical contrast imaging (dynamic)	- Tumor areas demonstrated a reduced lifetime in fluorescence compared with surrounding tissue; this was confirmed with a comparison with hematoxylin and eosin staining.	Novel imaging modality, with the goal of improving on FLIM by offering scalable data mapping
[46]	Eggert et al. (2022)	Prospective (98)	Oropharynx, larynx, hypopharyngeal	Hyperspectral imaging	- The 3D spatiospectral Densenet classification method has an average accuracy of 81%, a sensitivity of 83%, and a specificity of 79%	Noninvasive, label-free, accurate detection of malignant from healthy tissue
[47]	Stubbs et al. (2019)	Prospective (14)	Oropharynx and salivary gland	Free ICG-near-infrared fluorescent dye imaging (NIR)	- NIR imaging with ICG as a feasible option when infusion is performed the day prior to surgery, as 86% of primary tumors showed marked fluorescence - ICG imaging identified tumors in 3 cases that the surgeon was unable to visibly identify	Provides temporal data regarding optimal ICG dosing and demonstrates benefit in locating both primary tumors and sentinel nodes
[48]	Scott-Wittenborn et al. (2018)	Prospective (6)	Oropharynx	Free ICG-near-infrared fluorescence imaging	- ICG dye fails to help surgeons differentiate between normal and cancerous mucosa. Study was dropped after 6th patient because of the negative results	ICG may not be effective in head and neck cancers due to increased vasculature
[49]	Schmidt et al. (2016)	Prospective (55)	Oral cavity, larynx, oropharynx, hypopharynx	Free ICG-near-infrared fluorescent dye imaging (NIR)	- ICG positivity was associated with a 90.5%, 90.9% and 89.1% sensitivity, specificity, and accuracy, respectively. There were no adverse effects encountered.	This modality was demonstrated to be safe, feasible, and helpful when differentiating malignant from healthy tissue
[50]	Pan et al. (2020)	Prospective (20)	Oral cavity	Free ICG-near-infrared fluorescent dye imaging (NIR)	- Fluorescence was detected in all primary tumors in included patients. Abnormal fluorescence was detected in 4 patients, 2 of which were determined to have residual malignancy	The findings emphasize the utility of using ICG in margin determination before resection, as well as the tumor bed
[53]	Zhou et al. (2022)	Open-label phase I/II clinical trials (31)	Head and neck (HNSCC), high-grade glioma (HGG), lung adenocarcinoma (LAC)	Panitumumab-IRDye800- tagged near-infrared fluorescent images using Novadaq (open-field)	- NIR imaging enhanced tissue- contrast 5.2-, 3.4-, and 1.4-folds for HGG, HNSCC, and LAC, respectively, compared with WLE - The system detected positive or close margins with a 97% and 78% success rate in HNSCC and LAC, respectively, while 93% of HGG infiltrative edges with greater than 50% tumor cell density were detected.	NIR may be used with white light endoscopy in the detection of head and neck cancers. This may be performed at a higher fidelity compared with other tumors (HGG)
[51]	van Keulen et al. (2019)	Prospective (14)	Head and neck SCC (cutaneous and mucosal)	Panitumumab-IRDYE800CW- tagged near-infrared fluorescence imaging	- Fluorescence imaging improved surgical decision making in 3 cases (21.4%)	NIR may help define the primary tumor from surrounding mucosa
[52]	van Keulen et al. (2019)	Prospective (20)	Head and neck SCC (cutaneous and mucosal)	Panitumumab-IRDYE800CW- tagged near-infrared fluorescence imaging	- Tumors could clearly be imaged in situ, and imaging had a strong predictive value	Helpful with irregularly defined tumors, reduced positive margin rate
[54]	Steinkamp et al. (2021)	Prospective (13)	Oral cavity	ONM-100-ICG-tagged infrared fluorescence imaging	- Four intraoperative in vivo lesions were fluorescent, leading to the biopsy of 3 true positive cases and 1 false positive.	Potential for ONM-100 in malignant tissue identification in the context of metabolic acidotic tissue
[55]	Voskuil et al. (2020)	Prospective (13 HNC)	Head and neck SCC, breast, esophageal, colorectal	ONM-100-ICG-tagged infrared fluorescence imaging	- This study identified tumor-positive margins with 100% sensitivity, although specificity for HNCs was unclear due to pooling of findings	Safe, acid-dependent fluorescence that helps identify hypoxic, acidotic malignant tissues vs. healthy tissues

## Appendix A. Database Search Parameters

PubMed: ((“head and neck”(All Fields) OR “salivary gland”(All Fields) OR “oral”(All Fields) OR “oral cavity”(All Fields) OR “oral cavity”(All Fields) OR “nasopharyngeal”(All Fields) OR “oropharyngeal”(All Fields) OR “pharyngeal”(All Fields) OR “laryngeal”(All Fields)) AND (“cancer”(All Fields) OR “malignancy”(All Fields) OR “neoplasm”(All Fields)) AND “intraoperative imaging”(All Fields)) AND (english(Filter))
CINAHL: (“head and neck” OR “salivary gland” OR “oral” OR “oral cavity” OR “oral cavity” OR “nasopharyngeal” OR “oropharyngeal” OR “pharyngeal” OR “laryngeal”) AND (“cancer” OR “malignancy” OR “neoplasm”) AND (“intraoperative imaging”)
Web of Science: (“head and neck” OR “salivary gland” OR “oral” OR “oral cavity” OR “oral cavity” OR “nasopharyngeal” OR “oropharyngeal” OR “pharyngeal” OR “laryngeal”) AND (“cancer” OR “malignancy” OR “neoplasm”) AND (“intraoperative imaging”)
